# Palmitoylethanolamide in the Treatment of Failed Back Surgery Syndrome

**DOI:** 10.1155/2017/1486010

**Published:** 2017-08-10

**Authors:** Antonella Paladini, Giustino Varrassi, Giuseppe Bentivegna, Sandro Carletti, Alba Piroli, Stefano Coaccioli

**Affiliations:** ^1^Department of MESVA, School of Medicine, University of L'Aquila, L'Aquila, Italy; ^2^Paolo Procacci Foundation, Rome, Italy; ^3^Department of Internal Medicine, Rheumatology and Medical Pain Therapy, Perugia University School of Medicine, Terni, Italy; ^4^Department of Neurosurgery, “Santa Maria” General Hospital, Terni, Italy

## Abstract

**Introduction:**

This observational study was designed to evaluate the efficacy of ultramicronized palmitoylethanolamide (um-PEA) (Normast®) administration, as add-on therapy for chronic pain, in the management of pain-resistant patients affected by failed back surgery syndrome.

**Methods:**

A total of 35 patients were treated with tapentadol (TPD) and pregabalin (PGB). One month after the start of standard treatment, um-PEA was added for the next two months. Pain was evaluated by the Visual Analogue Scale (VAS) at the time of enrollment (*T*_0_) and after one (*T*_1_), two (*T*_2_), and three (*T*_3_) months.

**Results:**

After the first month with TDP + PGB treatment only, VAS score decreased significantly from 5.7 ± 0.12 at the time of enrollment (*T*_0_) to 4.3 ± 0.11 (*T*_1_) (*p* < 0.0001); however, it failed to provide significant subjective improvement in pain symptoms. Addition of um-PEA led to a further and significant decrease in pain intensity, reaching VAS scores of 2.7 ± 0.09 (*T*_2_) and 1.7 ± 0.11 (*T*_3_, end of treatment) (*p* < 0.0001) without showing any side effects.

**Conclusions:**

This observational study provides evidence, albeit preliminary, for the efficacy and safety of um-PEA (Normast) as part of a multimodal therapeutic regimen in the treatment of pain-resistant patients suffering from failed back surgery syndrome.

## 1. Introduction

Failed back surgery syndrome (FBSS) can be defined as “surgical end stage after one or several operative interventions on the lumbar neuroaxis, indicated to relieve lower back pain, radicular pain, or the combination of both without positive effect” [1]. Clinical presentation is characterized as a chronic pain syndrome which severely impacts the quality of a patient's professional and personal life. Typically, symptoms associated with FBSS include diffuse, dull, and aching pain, sharp, pricking pain involving the back and legs, and stabbing pain in the extremities due to abnormal sensibility. Several factors can contribute to the onset or development of FBSS including, but not limited to, either residual or recurrent disc herniation, persistent postoperative nerve root pressure, altered joint mobility, axial hypermobility with instability, scar tissue and fibrosis, depression, anxiety, and spinal muscular pain. An individual's predisposition to the development of FBSS might be due to systemic disorders such as diabetes, autoimmune disease, and peripheral vascular disease [2, 3]. Although the etiology, underlying mechanisms, and pathoanatomic correlations can differ greatly across cases of FBSS, there is a consensus that this syndrome is typically “mixed,” inasmuch as there are both nociceptive and neuropathic mechanisms responsible for pain [2, 4]. Treatment of FBSS includes a wide range of therapeutic options such as pharmacologic agents, physical therapy, behavioral medicine, transcutaneous electrical nerve stimulation, minor nerve blocks, and pulsed electromagnetic therapy [5]. The objectives of management should be directed to restoration of functional ability, improvement of quality of life, coping strategies, and pain self-management [2, 6]. A stereotyped approach is unlikely to succeed since each patient deserves individual consideration for management [2]. Therefore, it is important for physicians who treat this population to expand their knowledge of FBSS etiologies with appropriate diagnostic modalities [7]. Pharmacologic treatment is the first-line therapy for pain management as a conservative measure when surgery fails to provide significant improvement [8]. Treatment include antiepileptics, nonsteroidal anti-inflammatory drugs, oral steroids, antidepressants, and opioids. Antiepileptics, such as pregabalin (PGB), are widely used to treat the neuropathic component of pain in FBSS and may play a role in preventing pain after surgery [9, 10]. Chronic opioid use is associated with a multitude of side effects, including immunosuppression, androgen deficiency, constipation, and depression [8]. Tapentadol (TPD), a new centrally acting analgesic with two mechanisms of action (*µ*-opioid receptor agonism and noradrenaline reuptake inhibition), showed efficacy similar to classical opioids with better tolerability [11].

An important development in pain management has been the discovery that initiation and maintenance of neuropathic pain involve communication between neurons and nonneuronal immunocompetent cells, such as mast cells and microglia, together with a cascade of pro- and anti-inflammatory cytokines [12–14]. Mast cells are often found close to nociceptive nerve terminals when activated after nerve injury and release mediators that cause peripheral sensitization and enhanced responsiveness of central nervous system neurons [15]. The persistent and aberrant excitability of primary sensory ganglia might also activate spinal microglia and thereby propagate neuroinflammation, prolonging the inflammatory state and leading to a condition of chronic pain [16].

An innovative approach in the management of chronic pain is represented by palmitoylethanolamide (PEA), a member of the N-acylethanolamine family of fatty acid amide signaling molecules. PEA has an established history of antiallodynic and antihyperalgesic effects, which are mediated by downmodulation of proinflammatory mediator release from mast cells [17, 18] and reducing mast cell [19] and microglial cell activation [19, 20]. At the molecular level, PEA is a peroxisome proliferator-activated receptor alpha (PPAR-*α*) ligand that exerts anti-inflammatory, analgesic, and neuroprotective actions [21, 22]. Further, in a chronic constriction injury model of peripheral neuropathy, PEA's ability to rescue the peripheral nerve from inflammation and structural derangement was lost in PPAR-*α* null mice [23]. Several clinical studies have reported the use of ultramicronized PEA (um-PEA) in the treatment of various syndromes associated with chronic pain that are poorly responsive to standard therapies [24–26]. The ultramicronization process is often used in the preparation of pharmaceuticals, as it yields a crystalline structure with higher energy content and smaller particle size which contributes to better distribution and diffusion and therefore a greater pharmacological efficacy [27, 28]. Interestingly, a recent study reported that micronized PEA/um-PEA displayed better oral efficacy compared to nonmicronized PEA in a rat model of inflammatory pain [29]. Based on these observations, the present study was designed to evaluate the efficacy of um-PEA (Normast) add-on therapy in conjunction with TPD + PGB standard treatment in the management of chronic pain in pain-resistant patients suffering from FBSS.

## 2. Materials and Methods

**Figure 1:**
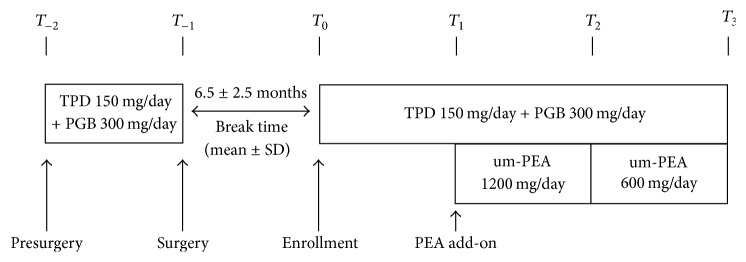
Study and treatment flow chart. The interval between each scheduled follow-up is one month. Break time is the mean time that passed after surgery, when patients return to the Out-Patient Center of Rheumatology and Pain Therapy complaining of persistent and increased pain. TPD: tapentadol; PGB: pregabalin; um-PEA: ultramicronized palmitoylethanolamide; SD: standard deviation.

This observational study was carried out at the Out-Patient Center of Rheumatology and Pain Therapy (Santa Maria General Hospital of Terni, Italy), affiliated to the University of Perugia Medical School. Patients selected for the study were suffering from FBSS caused by laminectomy, discectomy, or vertebral stabilization, who came to our attention complaining of an increase in pain intensity compared to the immediate postoperative condition. See Table 1 for patient demographics. Pain treatment and pain intensity evaluation on the Visual Analogue Scale (VAS) (before and immediately after surgery) were collected for all patients from their clinical charts. The VAS is a continuous scale comprised of a horizontal line, 10 centimeters (100 mm) in length, anchored by 2 verbal descriptors, one for each symptom (0 = no pain; 10 = the worst pain imaginable) [30]. Patients were treated with TPD and PGB at variable doses, for three months in this study. One month after the start of standard treatment, um-PEA (Normast, Epitech Group SpA, Saccolongo, Italia) was added at 1200 mg/day (two 600 mg tablets daily) for one month followed by 600 mg/day for the next month. Patients selected for this study were already under treatment with TPD + PGB in the month before surgery, with a mean dosage of 150 mg and 300 mg, respectively; the same dosages depending on the need of the patient were used in the prospective study. VAS evaluation was carried out every month for all patients at the time of enrollment (*T*_0_) and after one (*T*_1_), two (*T*_2_), and three (*T*_3_) months (Figure 1). This study was carried out in accordance with the Helsinki Declaration of 1964 and its subsequent revisions and Good Clinical Practice. All patients provided informed written consent to participate. Statistical analysis was carried out to evaluate mean differences along time. Gender, time, and time-gender interaction were used as covariates. Single comparisons were evaluated using the Tukey–Kramer adjusted test.

## 3. Results

**Figure 2:**
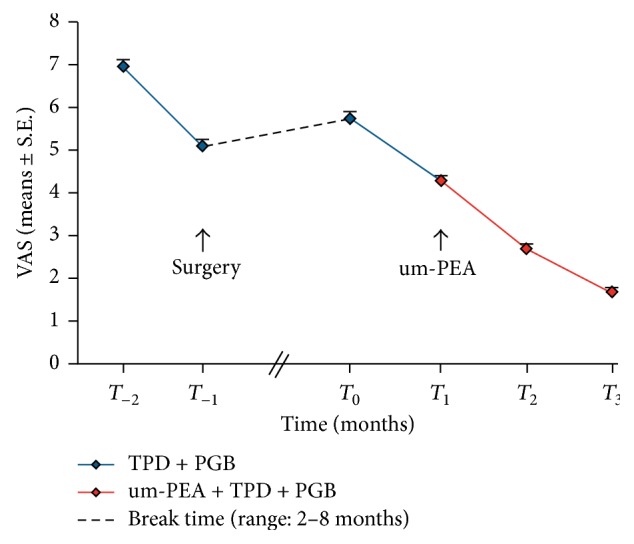
Changes in pain intensity by VAS measurement. FBSS patients selected at baseline (*T*_0_) received standard treatment comprising TPD + PGB for one month before surgery and showed a significant reduction immediately after surgery (*p* < 0.0001). There was a significant increase in pain intensity after a mean time of 6.5 ± 2.5 months (range: 2–8 months) following surgery (*p* = 0.0054). At this time (*T*_0_), patients were again given TPD + PGB for 3 months (up to *T*_3_); um-PEA was added for the last two months (*T*_1_ to *T*_3_). VAS reduction was statistically significant at each time (*p* < 0.0001).

Thirty-five patients were enrolled in this study, all having undergone a previous surgical procedure (demographic details are summarized in Table 1). All subjects received, in the month before surgery, a standard treatment comprising TPD + PGB, at the mean daily dose of 150 mg and 300 mg, respectively (individual patient dosing was determined by the physician, based on need). The mean intensity of pain evaluated by VAS one month before surgery (*T*_−2_) was 6.9 ± 0.14 and decreased significantly immediately after surgery (*T*_−1_) to 5.1 ± 0.13 (*p* < 0.0001). Subjects came to our attention after a median time of 6.5 ± 2.5 months (range: 2–8 months) after surgery and presented a considerable increase in the mean pain intensity at the time of enrollment, with a mean VAS score of 5.7 ± 0.12 (*p* = 0.0054) (Tables 2 and 3). All patients were treated for three months with standard medications (TPD + PGB) at mean daily doses of 150 mg and 300 mg, respectively. One month after the start of standard treatment, um-PEA (Normast) was added for two months: 1200 mg/day for the first month and 600 mg/day for the second month. During the first month with TDP + PGB treatment only, the VAS score decreased from 5.7 ± 0.12 at the time of enrollment (*T*_0_) to 4.3 ± 0.11 (*T*_1_); in the time periods following addition of um-PEA, VAS scores showed further decreases to 2.7 ± 0.09 (*T*_2_) and 1.7 ± 0.11 (*T*_3_, end of treatment) (Table 2, Figure 2). At each evaluation time, VAS was significantly reduced compared to the previous follow-up time (*p* < 0.0001) (Table 3).

## 4. Discussion

The present observational study provides preliminary evidence suggesting that um-PEA (Normast) as add-on treatment to conventional pharmacological regimens in patients suffering from FBSS contributes to a significant pain intensity reduction. As the complex physiopathology of this pain syndrome often renders monotherapy inadequate to achieve meaningful pain relief, polytherapy may thus represent a more fruitful therapeutic approach. TPD, a dual *µ*-opioid receptor agonist and noradrenaline reuptake inhibitor, is efficacious in patients with nociceptive and neuropathic low back pain, either alone [31, 32] or in combination with the anticonvulsant PGB, the latter acting as an agonist of high-voltage-activated calcium channels [33, 34]. The combination of TPD and PGB has a synergic effect in a rat model of neuropathic pain [35]. In the present study, TPD + PGB was administered as standard treatment to patients suffering from FBSS one month preceding surgery and after surgery (after a median time of 6.5 ± 2.5 months), when the patient first came to our clinic with complaints of persistent pain and pain of increased intensity. Although this conventional therapy significantly reduced VAS score, it failed to provide meaningful pain relief. Notably, pain reduction obtained in the first month after enrollment (*T*_1_ − *T*_0_) of 1.48 ± 0.14 was comparable to that achieved in the month leading up to surgery (*T*_−2_ − *T*_−1_) (1.83 ± 0.17), even though the latter period encompassed the surgery variable. While encouraging, the VAS score at *T*_1_ exceeded 4, an indication still of moderate pain intensity. In the search for new molecules as add-on therapy in the treatment of FBSS, we decided to assess the potential of um-PEA. PEA is an endogenous fatty acid amide signaling molecule produced on demand in response to cellular stress or injury. The anti-inflammatory and analgesic effects of PEA are likely accounted for by several not mutually exclusive mechanisms. PEA acts by downregulating mast cell degranulation via an “autacoid local inflammation antagonism” (ALIA) effect [36]. A “receptor mechanism” has also been proposed, based on the capability of PEA to directly stimulate either an as-yet uncharacterized cannabinoid CB2 receptor-like target [37, 38] or the nuclear peroxisome proliferator-activated receptor-*α*, the latter mediating many of PEA's anti-inflammatory effects [22]. In vivo studies show PEA to possess anti-inflammatory and pain-relieving properties [23, 37, 39]. Moreover, a number of clinical studies point to the potential therapeutic utility of this fatty acid amide in different neuropathic pain syndromes [24, 40–42]. The combination of PEA in association with other molecules results in pain reduction in neuropathic pain patients, with good safety and tolerability. At present, very little information is available concerning the use of PEA in FBSS. Gatti et al. [24] evaluated um-PEA's effects on chronic pain associated with different pathological conditions, including a group of 76 patients afflicted with FBSS. In their study, um-PEA's effect on reduction of pain intensity was evident for FBSS patients, as well as for the other groups of patients analyzed separately.

After the first month of TPD + PGB treatment, FBSS patients had a pain reduction of 1.48 ± 0.14 (*T*_1_ − *T*_0_). In the subsequent two months with um-PEA as add-on therapy, there was a further and significant decrease in pain intensity of 1.62 ± 0.15 (*T*_2_ − *T*_1_) after the second month and 0.97 ± 0.13 (*T*_3_ − *T*_2_) after the third month (Tables 2 and 3, Figure 2). To assess whether or not the increased effectiveness of therapy in the second month was attributable to um-PEA, we compared our results with those from an arm of a recent double-blind study where patients affected by chronic low back pain were treated with a combination of TPD (300 mg) + PGB (300 mg) for 2 months (after a titration period). Pain intensity assessed as VAS score decreased from 5.9 ± 0.10 (the reported VAS ± SE was calculated, following the system suggested in Figure  3 of Baron et al., 2015 [33]) at baseline (randomization time) to 4.4 ± 0.151 after one month of treatment and to 4.2 ± 0.201 after the second month, suggesting a decrease in TPD + PGB effect over time in this patient group [33]. One might well compare the two trends over time, as the starting VAS scores were similar both in our study and in that of Baron et al. [33] (5.7 and 5.9, resp.). In their comparative study, Baron et al. [33] observed decreased effectiveness of TPD + PGB therapy over time especially after the second month, with stabilization of the VAS score which did not decrease under a moderate score equal to 4. In contrast, our study demonstrated in the second month a clear increase in effectiveness of treatment, which led to a further and significant reduction in pain intensity that we ascribe to um-PEA add-on. The increased effectiveness of TPD + PGB treatment in the second month is unlikely to have occurred spontaneously, taking into account also the pain intensity trend curve of TPD + PGB combination only. Importantly, none of the patients experienced adverse events after um-PEA add-on to the standard treatment. The open-label design of this study, together with the limited number of patients, does not allow one to judge the extent to which um-PEA further improved the painful symptoms compared to standard treatment only. Furthermore, the relatively short treatment period (two months) does not allow one to predict effectiveness over the longer term. These caveats notwithstanding, our study is an example of how one may achieve an overall improvement in conventional drug treatment without side effects. The use of um-PEA (Normast) as add-on therapy might result in more efficacious pain relief through an action on immune cells, especially in cases refractory to standard therapies which act on neurons. Future studies should evaluate the benefits of combining these treatments on larger populations in controlled trials with more refined inclusion/exclusion criteria and conditions.

## Figures and Tables

**Table 1:** Patients' demographic and medical information.

	All	Male	Female
*Number of patients*, *n* (%)	35	15 (42.9)	20 (67.1)
*Mean age ± SD*	51.9 ± 14.7	49.3 ± 15.6	53.8 ± 14.1
*Surgical interventions*, *n* (%)			
Laminectomy	5 (14.3)	3 (20.0)	2 (10.0)
Discectomy	24 (68.6)	12 (80.0)	12 (60.0)
Vertebral stabilization	6 (17.1)	0	6 (30.0)
*Comorbidities* ^*∗*^, *n* (%)			
Hypertension	12 (34.3)	5 (33.3)	7 (35.0)
Obesity	10 (28.6)	3 (20.0)	7 (35.0)
Osteoarthritis	10 (28.6)	5 (33.3)	5 (25.0)
Chronic obstructive pulmonary disease	3 (8.6)	0	3 (15.0)
Chronic ischemic cardiomyopathy	2 (5.7)	1 (6.7)	1 (5.0)
None	9 (25.7)	5 (33.3)	4 (20.0)

^*∗*^Total is not 35 (100%) because some patients may present more comorbidities.

**Table 2:** Pain intensity by VAS measurement.

	*T*_−2_	*T*_−1_	*T*_0_	*T*_1_	*T*_2_	*T*_3_
VAS (mean ± SE)	6.9 ± 0.14	5.1 ± 0.13	5.7 ± 0.12	4.3 ± 0.11	2.7 ± 0.09	1.7 ± 0.11

VAS: Visual Analogue Scale; SE: standard error.

**Table 3:** Statistical analysis.

		Estimated difference	SE	*p* value
Presurgery (*T*_−2_)	Surgery (*T*_−1_)	1.833	0.166	<0.0001
Surgery (*T*_−1_)	Enrollment (*T*_0_)	−0.683	0.170	0.005
Enrollment (*T*_0_)	*T*_1_	1.483	0.144	<0.0001
*T*_1_	*T*_2_	1.625	0.149	<0.0001
*T*_2_	*T*_3_	0.975	0.129	<0.0001
